# Sculpting humoral immunity through dengue vaccination to enhance protective immunity

**DOI:** 10.3389/fimmu.2012.00334

**Published:** 2012-11-08

**Authors:** Wayne D. Crill, Holly R. Hughes, Nicole B. Trainor, Brent S. Davis, Matt T. Whitney, Gwong-Jen J. Chang

**Affiliations:** Arboviral Diseases Branch, Division of Vector-Borne Diseases, Centers for Disease Control and Prevention, Public Health Service, U.S. Department of Health and Human ServiceFort Collins, CO, USA

**Keywords:** dengue, dengue vaccines, DNA vaccines, immune refocusing, vaccine safety, antibody-dependent enhancement, original antigenic sin, dengue hemorrhagic fever

## Abstract

Dengue viruses (DENV) are the most important mosquito transmitted viral pathogens infecting humans. DENV infection produces a spectrum of disease, most commonly causing a self-limiting flu-like illness known as dengue fever; yet with increased frequency, manifesting as life-threatening dengue hemorrhagic fever (DHF). Waning cross-protective immunity from any of the four dengue serotypes may enhance subsequent infection with another heterologous serotype to increase the probability of DHF. Decades of effort to develop dengue vaccines are reaching the finishing line with multiple candidates in clinical trials. Nevertheless, concerns remain that imbalanced immunity, due to the prolonged prime-boost schedules currently used in clinical trials, could leave some vaccinees temporarily unprotected or with increased susceptibility to enhanced disease. Here we develop a DENV serotype 1 (DENV-1) DNA vaccine with the immunodominant cross-reactive B cell epitopes associated with immune enhancement removed. We compare wild-type (WT) with this cross-reactivity reduced (CRR) vaccine and demonstrate that both vaccines are equally protective against lethal homologous DENV-1 challenge. Under conditions mimicking natural exposure prior to acquiring protective immunity, WT vaccinated mice enhanced a normally sub-lethal heterologous DENV-2 infection resulting in DHF-like disease and 95% mortality in AG129 mice. However, CRR vaccinated mice exhibited redirected serotype-specific and protective immunity, and significantly reduced morbidity and mortality not differing from naїve mice. Thus, we demonstrate in an *in vivo* DENV disease model, that non-protective vaccine-induced immunity can prime vaccinees for enhanced DHF-like disease and that CRR DNA immunization significantly reduces this potential vaccine safety concern. The sculpting of immune memory by the modified vaccine and resulting redirection of humoral immunity provide insight into DENV vaccine-induced immune responses.

## INTRODUCTION

Dengue viruses (DENV) are the most prevalent arthropod-borne viral pathogens infecting humans. These mosquito-transmitted viruses, members of the *Flaviviridae*, are endemic in most tropical and sub-tropical countries with nearly half of the world’s population living at risk of DENV infection and resulting in over a million estimated infections annually (Gubler, 2002; Mackenzie et al., 2004). Infection with DENV can cause a broad range of symptoms, ranging from subclinical, to the self-limiting flu-like illness dengue fever, to the more severe and life-threatening dengue hemorrhagic fever and shock syndrome (DHF/DSS), characterized by increased vascular permeability producing plasma leakage, severe thrombocytopenia and hypotension leading to circulatory collapse (Gubler, 2006). DENV prevalence, infection rates, and disease severity have increased exponentially since the middle of the last century (Guzman et al., 2010). Despite decades of interest, need, and effort there remains no available dengue vaccine (Guy et al., 2010; Miller, 2010; Murphy and Whitehead, 2011; Thomas, 2011).

Dengue vaccine development faces both biological and immunological challenges. These include the necessity for a multivalent vaccine inducing balanced immunity, the lack of an animal model for DENV disease, and concerns regarding vaccine-induced immunopathology (Murphy and Whitehead, 2011; Heinz and Stiasny, 2012). DENV immune responses are both protective and pathogenic and this duality challenges vaccine development (Rothman, 2004). There are four closely related yet phylogenetically distinct DENV serotypes (DENV-1, -2, -3, and -4) and infection with any one serotype appears to induce life-long serotype-specific immunity yet cross-protection between serotypes is limited and transient (Sabin, 1952; Kuno, 2003). Thus, in endemic regions individuals may be susceptible to up to four different DENV infections. Although there are a number of risk factors associated with DHF such as virus and host genetics, the strongest risk factor for severe dengue pathology is secondary infection with a previously unencountered (heterologous) serotype (Murphy and Whitehead, 2011). This association explains the exponential increase of DHF/DSS in recent decades as co-circulation and simultaneous transmission of the four DENV serotypes increases both temporally and geographically (Mackenzie et al., 2004; Gubler, 2006; Guzman et al., 2010). In humans, increasing viral load correlates with DENV disease severity and a large body of evidence points to the importance of immune enhancement being a causal factor for the increased viral loads associated with DHF (Vaughn et al., 2000; Libraty et al., 2002).

Two distinct yet not mutually exclusive mechanisms of immune enhancement explain the pathogenic manifestations characterizing DHF/DSS. The leading hypothesis is that DHF occurs via antibody-dependent enhancement (ADE) of infection, a phenomenon originally described for flaviviruses but later found to occur with diverse pathogens (Hawkes, 1964; Halstead, 1989; Morens, 1994; Thomas et al., 2006). Preexisting cross-reactive antibodies from a previous infection (or maternal antibodies in infants) recognize and bind to heterologous virus in a secondary infection (primary infection in infants), yet are unable to neutralize this virus, either because they are non-neutralizing, or for a lack of sufficient avidity or occupancy (Pierson et al., 2008). However, these non-neutralizing antibody–virus complexes increase the infection of monocytes via their Fc receptors, dramatically increasing viral replication and load, thereby causing DHF. Weakly- and non-neutralizing cross-reactive antibodies induced from immunodominant B cell epitopes are known to comprise the majority of the humoral immune response to DENV infection (Lai et al., 2008; Crill et al., 2009). In a recently developed type I/type II IFN receptor knock-out mouse model (AG129) of DENV disease (Williams et al., 2009), sub-neutralizing levels of homologous or heterologous DENV immune mouse sera (Balsitis et al., 2010; Zellweger et al., 2010) or cross-reactive human monoclonal antibodies (MAbs; Beltramello et al., 2010) enhanced viral replication *in vivo* producing a DHF-like disease.

The second, related mechanism of immune enhanced DENV pathology, also a generalized phenomenon, posits that there is a highly skewed cellular response to heterologous DENV infection driven by low affinity, cross-reactive memory CD4+ and CD8+ T cells (Brehm et al., 2002; Welsh and Selin, 2002; Welsh et al., 2004). Cross-reactive T cell epitopes have been identified across the DENV proteome, however, immunodominant CD8+ T cell epitopes in non-structural protein NS3 appear to be those most strongly associated with DHF (Mathew and Rothman, 2008; Duangchinda et al., 2010; Friberg et al., 2011).

The common theme throughout DENV immune enhancement is the concept of “original antigenic sin” describing a phenomenon first observed in response to influenza, it has since been found to be common across diverse taxa (Francis, 1953; Welsh and Fujinami, 2007). Original antigenic sin describes the shift in the immunodominance hierarchy that occurs when prior exposure to cross-reactive antigens alters and inhibits subsequent immune response to related antigens, either in new infections or through vaccination (Brehm et al., 2002). Both humoral and cellular responses can be plagued by such misdirected or inappropriate heterotypic immunity (Welsh and Fujinami, 2007). The phenomenon is most severe when the cross-reactive antigenic responses are immunodominant as is the case with both cellular and humoral DENV immunity. Thus, for DENV, not only does original antigenic sin appear to play an important role in the more severe DENV pathologies but it also creates a potential vaccine safety issue as a lack of protective tetravalent vaccine-induced immunity could prime vaccinees for DHF upon subsequent infection (Durbin and Whitehead, 2011; Murphy and Whitehead, 2011; Schmitz et al., 2011; Heinz and Stiasny, 2012).

Concerns for dengue vaccination priming individuals for severe disease via immune enhancement have necessitated that dengue vaccines must produce balanced, tetravalent, and protective immunity, optimally derived from a single dose vaccination providing long-lasting protection (Miller, 2010; Murphy and Whitehead, 2011; Heinz and Stiasny, 2012). Inducing balanced and protective immunity has been the biggest hurdle in DENV vaccination (Sun et al., 2009; Morrison et al., 2010; Durbin and Whitehead, 2011). Tetravalent live-attenuated vaccines are susceptible to replication interference between different vaccine viruses (Guy et al., 2009) requiring multiple boosts spread over 6–12 months (Sin Leo et al., 2012). Nevertheless, multiple live-attenuated DENV vaccines are advancing in human clinical trials and promising results suggest potential licensure this decade (Durbin and Whitehead, 2009; Guy et al., 2011; Heinz and Stiasny, 2012).

We have developed a second generation flavivirus vaccine platform attempting to address some of the potential obstacles to DENV vaccine development. The DNA vaccine platform consists of an expression plasmid containing only the envelope (E, the primary protective antigen) and premembrane (prM) structural protein genes. Upon uptake by host cells the structural genes are transcribed and translated and the proteins self-assemble into virus-like particles (VLPs) presenting authentic E proteins (Chang et al., 2001, 2003). Our wild-type (WT) flavivirus DNA vaccines are demonstrably safe, immunogenic, and protective against Japanese encephalitis, West Nile, and dengue viruses in both non-human animals (Davis et al., 2001; Chang et al., 2003, 2007; Hughes et al., 2012b) and in humans (Martin et al., 2007). DNA vaccines stimulate strong CD4+ T cell responses as do inactivated and subunit vaccines and yet also strongly stimulate CD8+ T cell responses similar to live-attenuated vaccines (Laylor et al., 1999; Martin et al., 2007). Because VLPs lack infectious RNA and are non-replicating, multivalent DNA vaccines are less susceptible to replication interference than live-attenuated vaccines (Petersen and Roehrig, 2007; Guy et al., 2009). Most importantly, DNA vaccines can be readily manipulated and engineered to prime specific epitopes and redirect immunity. This sculpted immune memory priming can redirect subsequent vaccine boosts or natural exposure toward protective, DENV serotype-specific epitopes increasing both vaccine safety and efficacy (Miller, 2010; Nara et al., 2011; Schmitz et al., 2011; Hughes et al., 2012a).

In this work we introduce substitutions into two distinct E protein antigenic regions of a WT DENV-1 DNA vaccine (pVD1-WT) ablating cross-reactive, immunodominant, weakly or non-neutralizing B cell epitopes (Crill et al., 2009) associated with immune enhancement, to limit the ability of this cross-reactivity reduced vaccine (pVD1-CRR) to potentiate DHF via ADE. We extend upon previous proof in principle work with DENV-2 (Chang et al., 2003; Hughes et al., 2012a,b) to test pVD1-CRR safety and efficacy via active vaccination of AG129 mice. Both pVD1-WT and pVD1-CRR were equally protective against lethal, homologous DENV-1 challenge. pVD1-CRR vaccinated mice produced significantly less antibody recognizing immunodominant enhancing epitopes, exhibited significantly increased serotype-specific and protective immunity in response to heterologous DENV-2 challenge, and were less susceptible to lethally enhanced heterologous DENV-2 DHF-like disease. These results provide new insights into sculpting humoral immunity that should advance the development of DENV vaccines and our understanding of the mechanisms of their protective efficacy.

## MATERIALS AND METHODS

### VACCINE CONSTRUCTION, CHARACTERIZATION, AND PREPARATION

The construction of pVD1-WT and pVD1-CRR vaccine plasmids was the same as for DENV-2 DNA vaccine plasmids that have been previously described in detail (Chang et al., 2003; Crill et al., 2009). Briefly, pVD1-WT was constructed using DENV-1 strain (BC94/95) for cDNA cloning of prM and 80% DENV-1 E protein with the C-terminal 20% of E replaced with the homologous region of Japanese encephalitis virus (JEV). Eighty percent DENV-1 E corresponds to the ectodomain and the C-terminal 20% corresponds to the helical stems (E-H1, E-H2) and transmembrane domain (TMD) anchor helices (E-T1, E-T2; Zhang et al., 2003; Lin et al., 2011). The pVD1-WT and pVD1-CRR vaccines utilized in this study also contained a potent CD4+ T cell epitope identified in West Nile virus (WNV) and located in the TMD. The inclusion of this epitope into our DENV-2 DNA vaccine (pVD2) and into pVD2-CRR was shown to boost the neutralizing antibody responses of vaccinated mice about twofold. The identification, construction, and characterization of this epitope has recently been described in detail (Hughes et al., 2012b). Introduction of the WNV TMD CD4+ epitope into pVD1 required introducing four amino acid substitutions (V474I, A484T, T488V, and V493L) into the C-terminal 20% JEV E sequence of pVD1.

pVD1-CRR was constructed using the primers listed in **Table 1** and pVD1-WT as the DNA template with Stratagene Quick Change multi, site-directed mutagenesis kit (Stratagene, La Jolla, CA) following the manufacturer’s recommended protocols. Structural gene regions and regulatory elements of all plasmids were sequenced entirely upon identification of the correct mutation.

**Table 1:** Nucleotide sequences of mutagenic primers.

Primer	Primer Sequence (5′-3′)	Nucleotide substitution	Amino acid substitution
D1-G106R	TTCCTTTCCGAAGAG**A**C**G**ACAGCCATTGCCCCAGCC****	GGG-CGT	Gly-Arg
D1-L107D	TTCCTTTTCCGAA**ATC**CCCACAGCCATTGCCCCAG	CTC-GAT	Leu-Asp
D1-G106RL107D	CTTCCTTTTCCGAA**ATC**CC**G**ACAGCCATTGCCCCAGCC	GGA-CGG CTC-GAT	Gly-ArgLeu-Asp
D1-K310D	AGCCACTTC**G**T**C**CTCTAGCTTGAATGAGCCTGTGC	AAA-GAC	Lys-Asp
D1-E311K	TCAGCCAC**C**T**T**TTTCTCTAGCTTGAATGAGCCTGTGC	GAA-AAG	Glu-Lys
D1-K310DE311K	GGGTCTCAGCCAC**C**T**TG**T**C**CTCTAGCTTGAATGAGCCTGTGC	AAA-GAC GAA-AAG	Lys-AspGlu-Lys
D1-P364Q	GCCTCAATGTTGAC**CT**GTTTTTCTTTGTCAGTGACTATGGG	CCA-CAG	Pro-Gln

*Mutated nucleotides are shown in bold.*

### ETHICS STATEMENT

All animal experiments were approved by the IACUC at the US Centers for Disease Control and Prevention (CDC), Division of Vector-borne Disease (DVBD); an AAALAC certified facility. Protocols followed the US National Guidelines defined in the *Guide for the Care and Use of Laboratory Animals*, 8th edition, 2011, National Academy Press.

### MICE: PROTECTIVE EFFICACY

Type I/type II IFN receptor-knock-out mice (AG129) were obtained from the DVBD, CDC colony. Six-week-old AG129 mice (DVBD, *n* = 26) were immunized intramuscularly (i.m.) with 100 μg of pVD1-WT or pVD1-CRR vaccine (50 μg in each thigh) at 0, 4, and 8 weeks and with naїve age-matched controls (*n *= 8), were challenged at 12 weeks intraperitoneally (i.p.) with 1.1 × 10^5^ focus forming units (FFU) of DENV-1 Mochizuki strain. Based upon results from previous dose-titration experiments four animals from each vaccine treatment and two naїve mice were *a priori* selected for sacrifice 3 and 8 days post challenge (DPC). Sick animals reaching predetermined morbidity endpoints were anesthetized, total blood was collected via cardiac puncture, euthanized and carcasses were frozen at -80°C for subsequent analysis.

### MICE: VACCINE SAFETY

Six-week-old AG129 mice (DVBD, *n *= 26) were immunized with 100 μg of either pVD1-WT or pVD1-CRR vaccine (50 μg in each thigh, i.m.) at 0 weeks and with age-matched naїve mice (*n* = 10) were i.p. challenged at 12 weeks with 4.2 × 10^5^ FFU of DENV-2 S221 (gift from S. Shresta; Zellweger et al., 2010). Four mice from each vaccine treatment and two naїve mice were *a priori* selected for sacrifice 3 DPC. Sick animals reaching predetermined morbidity endpoints were anesthetized, total blood was collected via cardiac puncture, euthanized, and necropsy was performed with livers collected and stored in 10% formalin for histology.

### HISTOLOGY

Liver tissue sections were prepared, stained, and analyzed by Todd Bass at the Colorado State University (CSU) College of Veterinary Medicine and Biomedical Sciences Histology Laboratories. Hematoxylin and Eosin (H&E) staining and immunohistochemistry slides were interpreted by Dr. Barbara Powers at CSU and Dr. Nord Zeidner (CDC) assisted with H&E slide interpretation and photography. IHC utilized Ventana Medical Systems ultraView Universal Alkaline Phosphatase Red Detection Kit following the manufacturer’s recommended protocols and a Benchmark Ultra automated system. Pretreatment was with protease II for 6 min, rabbit anti DENV-2 NS1 serum, generated at the CDC, was used at a 1:20 dilution, and anti-rabbit secondary antibody was applied for 12 min.

### CHARACTERIZATION OF pVD1-CROSS-REACTIVITY REDUCED PLASMID VACCINE EXPRESSED VIRUS-LIKE PARTICLE ANTIGEN

Antigen-capture ELISA (Ag-ELISA) was used to characterize VLP antigen following previously described protocols (Crill et al., 2009). Briefly, VLP antigen was collected from mutagenized and WT pVD1 transformed COS-1 cells. Secreted antigen was captured in the inner 60 wells of Immulon II HB flat-bottom 96-well plates (Dynatech Industries, Inc., Chantilly, VA) with polyclonal rabbit anti-DENV-1 WT VLP sera, incubated overnight at 4°C, and wells were blocked with 300 μl of StartBlock blocking buffer (Pierce, Rockford, IL, USA) according to the manufacturer’s recommended protocol. Antigen was diluted twofold in PBS, incubated for 2 h at 37°C and detected with murine hyper-immune ascitic fluid (MHIAF) specific for DENV-2 diluted in 5% milk/PBS. MHIAF was detected using horseradish peroxidase (HRP) conjugated goat anti-mouse IgG (Jackson ImmunoResearch, Westgrove, PA) in 5% milk/PBS and incubated for 1 h at 37°C. Bound conjugate was detected with 3,3′5,5′-tetramethylbenzidine substrate (TMB; Neogen Corp., Lexington, KY), the reaction was stopped with 2N H_2_SO_4_ and measured at *A*_450_ using a Synergy HT Multi-Detection Microplate Reader (Bio-Tek Instruments, Inc., Winooski, VA). WT and mutant antigens were screened against the MAb panel using the same ELISA protocol as above with the exception that twofold dilutions of the specific MAb replaced the anti-DENV-2 MHIAF and antigens were used at a single standardized concentration producing an optical density (OD) of 1.0. Standardized concentrations of WT and mutant VLP antigens were analyzed in Ag-ELISA to determine MAb endpoint reactivities.

### MONOCLONAL ANTIBODIES

MAbs 4G2, 6B6C-1, 4A1B-9, 1B7, D3-5C9-1, 1A1D-2, 9D12, 9A3D-8, 3H5, and D2-1F1-3 were obtained from hybridomas in the collection of the Arboviral Diseases Branch, Division of Vector-borne Diseases, Centers for Disease Control and Prevention. Many of these MAbs originated from the work of John Roehrig (Roehrig et al., 1998), 4G2, 5H3, 1B7, D3-5C9-1, 9D12, 3H5, and D2-1F1-3 hybridomas were originally obtained by the CDC from the Walter Reed Army Institute (Henchal et al., 1985). MAbs 23-1, 23-2, and 5-2 were provided by Dr. L.-K. Chen of Tzu Chi University, Hualien, Taiwan. MAbs 10-D35A, 20-783-745014, and MDVP-55A are commercially available and were purchased from Fitzgerald Industries International, GenWay Biotech Inc., and Immunology Consultants Laboratory Inc. respectively.

### EPITOPE-SPECIFIC IgG ELISA

We used DENV-1 and DENV-2 epitope-specific IgG ELISA (ES-ELISA) to determine total DENV-1 or DENV-2 IgG endpoint titers and to determine percent DENV-1 or DENV-2 EDII_FP_ and non-EDII_FP_EDIII_CR_ epitope-specific IgG. The DENV-2 ES-ELISA has been previously described (Crill et al., 2009; Hughes et al., 2012a) and the DENV-1 ES-ELISA was similarly used with a few modifications. Briefly, Rabbit anti-pVD1 or pVD2 sera was coated onto plates overnight at 4°C. Standardized concentrations of WT and CRR antigens were captured, AG129 mouse serum was diluted two- to fourfold down the plate and detected with goat anti-mouse IgG (Jackson ImmunoResearch). The DENV-1 epitope-specific knock-out antigens utilized are described in **Table 2**. OD_450_ values were modeled as non-linear functions of the log_10_ serum dilutions using a non-linear sigmoidal dose-response (variable slope) regression in Graph Pad Prism version 4.0 (GraphPad Software, Inc. La Jolla, CA) and endpoint titrations were determined as the titer where the OD_450_ value equaled two-times the OD_450_ value of the test serum reacted against normal COS-1 antigen.

**Table 2:** MAb reactivities for DENV-1 VLP mutants^1^.

**MAb:**		MHAF	4G2	6B6C-1	4A1B-9	23-1	23-2	5H3	5-2	1B7	D3-5C9-1	10-D35A	20-783-74014	1A1D-2	9D12	MDVP-55A	D2-1F1-3
**CR^2^:**		Polyclonal	Group	Group	Group	Group	Group	Group	Sub Group	Sub Group	Complex	Complex	Complex	Sub Complex	Sub Complex	Sub Complex	Type specific
**Virus^3^:**		D1	D2	SLEV	MVEV	WNV	JEV	YFV	JEV	D2	D4	DENV	DENV	D2	D1	DENV	D1
	**Percent secretion**																
**VLP construct**																	
*WT DENV-1*^4^	*100*	*5.7*	*6.6*	*5.1*	*4.8*	*5.4*	*6.6*	*5.1*	*4.5*	*6.3*	*4.7*	*5.7*	*5.1*	*5.7*	*5.1*	*4.2*	*6.3*
G106R	200	100	**<0.1**	12.5	**1.5**	25	100	nd	100	**0.8**	100	nd	nd	25	100	nd	100
L107D	150	100	**<0.1**	50	100	**<0.1**	25	nd	**<0.6**	50	100	nd	nd	100	100	nd	100
G106RL107D	130	100	**<0.1**	**0.8**	**1.5**	**<0.1**	**<0.1**	**0.8**	**3.0**	100	100	nd	nd	100	50	nd	100
K310D	63	100	**<0.1**	100	100	100	100	nd	150	100	100	100	**<0.1**	**1.5**	**<0.8**	**<1.3**	100
E311K	50	100	100	100	100	100	100	nd	50	100	100	100	**3.9**	50	**<0.16**	**5.6**	100
P364Q	50	100	**<0.1**	100	100	100	100	nd****	50	100	100	100	50	100	100	100	100
K310DE311K P364Q	10	100	100	50	12.5	100	100	nd	50	100	100	100	**<0.1**	**<0.8**	**<0.16**	**<0.6**	100
G106RL107DK 310DE311K P364Q	91	100	**<0.1**	**3.0**	**<0.1**	**<0.1**	**<0.1**	**<0.1**	**<0.6**	100	100****	50	**<0.1**	**<0.1**	**<0.1**	**<0.6**	100

*nd, not determined.*

1 Reactivity levels for MAbs exhibiting varying cross-reactivity (CR) selected from different flaviviruses for wild-type (WT) and mutant VLP;

2 MHAF is polyclonal murine hyper-immune ascitic fluid, group CR antibodies recognize all viruses of at least the four major pathogenic flavivirus serocomplexes; sub-group CR MAbs recognize all or some members of two or more different flavivirus serocomplexes (e.g., MAb 5-2 recognizes JEV, DENV-1 and DENV-2 respectively); complex and sub-complex CR MAbs recognize all four DENV complex viruses or a subset thereof respectively, and type-specific MAbs recognize only DENV-1;

3 Virus the MAb was raised against; D1, dengue virus serotype 1 (DENV-1); D2, DENV-2; D3, DENV-3; D4, DENV-4; SLEV, St. Louis encephalitis virus; MVEV, Murray Valley encephalitis virus; WNV, West Nile virus; JEV, Japanese encephalitis virus; YFV. yellow fever virus. MAbs 20-783 and MDVP-55A are commercial MAbs raised against “dengue virus”

4 MAb reactivities for wild-type (WT) DENV-1 VLP are presented as inverse log_10_ Ag-capture ELISA endpoint values and all mutant VLPs as percent of remaining reactivity compared to WT. Emboldened values represent reactivity reductions greater than 90% relative to WT.

E protein structural domain II fusion peptide (EDII_FP_) and non-EDII_FP_ EDIII cross-reactive (EDII_FP_ EDIII_CR_) epitope-specific IgG percentages were calculated as previously described (Crill et al., 2009; Hughes et al., 2012a) with minor modifications. The DENV-1 epitope-specific IgG percentages for all vaccinated mice were calculated by dividing the IgG endpoint titer obtained with each knock-out antigen (EDII_FP_ or EDII_FP_EDIII_CR_) by the endpoint titer obtained with pVD1-WT antigen on the same sera, subtracting this value from 1.0, and multiplying by 100. Specifically, EDII_FP_ epitope-specific percentages were calculated as 100× [1.0-(DENV-1 EDII_FP_ antigen endpoint/ DENV-1 WT antigen endpoint)]. Non-EDII_FP_EDIII_CR_ IgG proportions were calculated as 100*(endpoint EDII_FP_EDIII_CR_/endpoint WT) for WT vaccinated mice and because pVD1-CRR vaccinated mouse sera contain antibodies that do not recognize some WT antigen epitopes (WT antigen acts as the knock-out antigen) but recognize the modified epitopes of the CRR antigens, the pVD1-CRR antigen (EDII_FP_EDIII_CR_) replaced pVD1-WT antigen to determine 100% E reactivity for pVD1-CRR vaccinated mice and similarly, the non- EDII_FP_EDIII_CR_ IgG proportion for pVD1-CRR immunized mice was calculated as 100*(DENV-1 WT antigen endpoint/ DENV-1 EDII_FP_EDIII_CR_ antigen endpoint). DENV-2 epitope-specific IgG populations were similarly determined but used the previously described DENV-2 EDII_FP_ and EDII_FP_EDIII_CR_ knock-out antigens to determine endpoints (Crill et al., 2009; Hughes et al., 2012a) for both pVD1-WT and pVD1-CRR vaccinated sera. In cases where the endpoint titer determined with a mutant antigen was the same or greater than the endpoint titer obtained with the cognate antigen we interpreted these cases as undetectable levels of antibody recognizing the epitope of interest and the percent of IgG was conservatively set at 1.0%. Calculated EDII_FP_ IgG endpoint titers were determined indirectly with the EDII_FP_ knock-out and WT antigens by multiplying the total IgG endpoint titer by the percent EDII_FP_ IgG.

### EPITOPE-BLOCKING ELISA

Epitope blocking ELISA was utilized to determine the vaccinated mouse response to well-characterized murine MAb epitopes. This ELISA was set up similar to ES-ELISA in that plates were coated overnight at 4°C with rabbit anti-DENV-2 serum, blocked with StartBlock (Pierce), dried and WT DENV-2 VLP antigen was captured 1 h at 37°C. After washing, pVD1-WT or pVD1-CRR vaccinated, DENV-2 challenged mouse serum (3 DPC, pooled for each vaccine treatment due to limited availability of serum) was diluted 1:40 in wash buffer and incubated 1hr at 37°C. Following pooled serum incubation and wash, 0.5 μg of MAb conjugated with HRP was added to each well and incubated for 1 h at 37°C to compete with the already bound antibody from vaccinated mouse serum for WT DENV-2 VLP antigen. Bound conjugate was detected with TMB substrate and plates incubated for 10 min prior to being stopped with H_2_SO_4_ and ODs were determined as OD = OD of A_450_ - OD of A_63__0_. Percent blocking was determined by comparison to replicate wells with 0.5 μg HRP labeled MAb competing against pre-adsorbed normal mouse serum using the following calculation, 100–100*[(OD of vaccinated serum on DENV-2 Ag-OD of vaccinated serum on normal Ag)/OD of normal serum on DENV-2 Ag-OD of normal serum on normal Ag)].

### *IN VITRO* ANTIBODY-DEPENDENT ENHANCEMENT

Heat inactivated pVD1-WT and pVD1-CRR vaccinated mouse sera (12 weeks post vaccination) were pooled into four groups for each vaccine treatment, diluted, and titrated. Virus (DENV-2 16681) was added to each dilution and incubated for 1 h at 37°C. K562 cells (MOI = 0.5) were added to the antibody–virus complexes and incubated 2 h. After infection, cells were centrifuged, supernatants removed, resuspended in RPMI media (10% FBS) and plated. DENV infection alone was used as virus control.

### FOCUS REDUCTION MICRO-NEUTRALIZATION ASSAY

Focus reduction micro-neutralization assay (FRμNT) assay was utilized as previously described (Crill et al., 2009) with few modifications. Vaccinated mouse sera were diluted 1:10, heat inactivated, titrated twofold to the volume of 40 μL, and 320 virus FFU/40 μL (DENV-1 56BC94/95, DENV-2 16681, DENV-3 116RC1396, or DENV-4 130) was added to each dilution. FRμNT titers were calculated for each virus relative to a back titration. Exact 50% of FRμNT titers were modeled using Graph Pad Prism version 4 sigmoidal dose response (variable slope) non-linear regression. Values are the average of two independent replicates.

### VIREMIA AND BRAIN VIRUS TITERS

Viremia was determined in a similar antigen-focus assay as described for FRμNT except that no virus was added. Cells were incubated, overlaid, acetone fixed, immunostained, and counted as described for FRμNT. Virus brain titers were determined from previously frozen mouse carcasses. Brain tissue was aspirated with a 3 mL syringe and 18 gage needle, weighed, and resuspended in 200 uL BA-1 media. Fifty microliters was used in the FRμNT as described. FFU/g of tissue was back calculated from the aspirated brain mass.

### STATISTICAL ANALYSIS

Non-parametric analyses used the Mann–Whitney *U* test and paired analyses utilized the paired *t*-test. All statistical analyses were performed using GraphPad Prism 4.02.

## RESULTS

### VACCINE CONSTRUCTION

**FIGURE 1:**
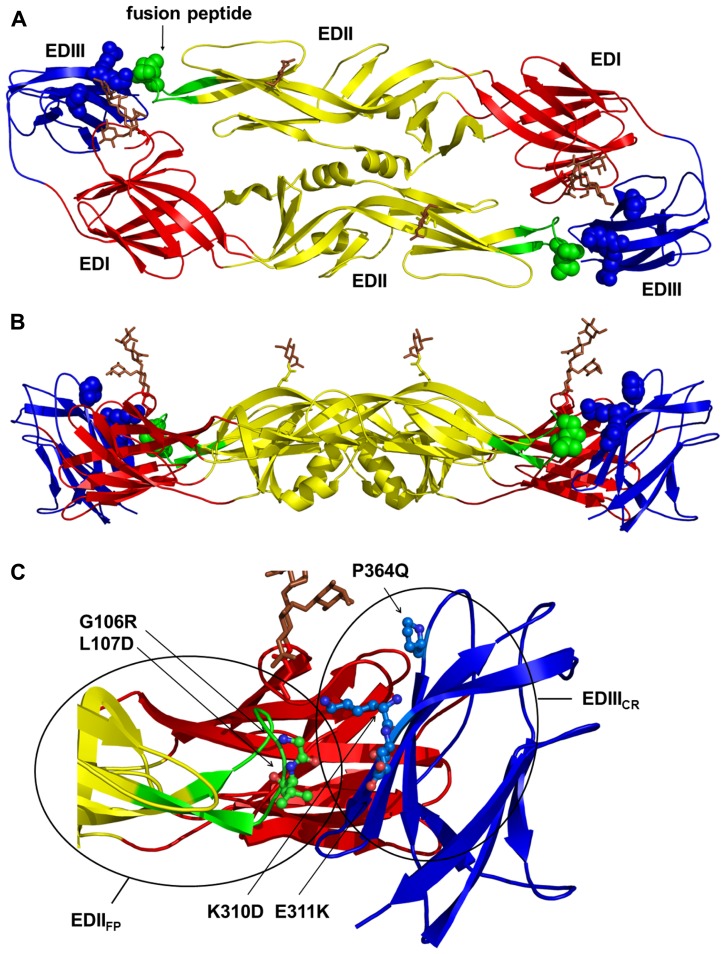
**Structural locations of the pVD1-CRR envelope (E) protein cross-reactive epitope knock out substitutions.** The locations of the DENV-1 E substitutions introduced to construct pVD1-CRR mapped on the crystal structure of the homologous DENV-2 E protein dimer (Modis et al., 2003). **(A)** Locations of pVD1-CRR substitutions (spheres) on a ribbon diagram of the mature E dimer as it appears looking straight down toward the virion surface. The three structural domains are labeled and colored red for E domain I (EDI), yellow for EDII, and blue for EDIII. The EDII fusion peptide (EDII_FP_) is also labeled and colored green. The glycans in EDI (N67) and EDII (N153) are depicted as stick representations and colored brown. **(B)** Side view of the E protein dimer with all colorations the same as in panel A. **(C)** Close up of the EDII_FP_ from one monomer and EDIII region of the other at the dimer interface, as it appears in panel B. The side chains of two residues in the EDII_FP_ and three residues in EDIII where CRR substitutions were introduced are depicted as ball and stick representations and labeled with the introduced substitutions. Structural domains are colored as in panel A and the two antigenic regions EDII_FP_ and EDIII_CR_ are noted with circles roughly representing the size of an IgG footprint binding to these regions.

Based upon previously published and unpublished results with DENV and other flaviviruses (Crill and Chang, 2004; Sukupolvi-Petty et al., 2007; Gromowski et al., 2008; Crill et al., 2009; Hughes et al., 2012a), we introduced specific substitutions into cross-reactive B cell epitopes of the envelope protein of a DENV-1 prM/E expression plasmid (Chang et al., 2003) to generate a DENV-1 DNA vaccine candidate with reduced ability to induce the cross-reactive antibodies that can be associated with ADE. The DENV-1 WT DNA vaccine (pVD1-WT) utilized as the template for the CRR vaccine (pVD1-CRR) in this study was based upon previous studies with DENV-2 vaccines and contains a potent CD4+ epitope identified in WNV and demonstrated to increase the immunogenicity of DENV-2 DNA vaccines (Hughes et al., 2012b). We introduced substitutions into two important cross-reactive regions of the DENV E protein, the highly conserved fusion peptide of structural domain II (EDII_FP_) and the A-strand and DE loop of domain III (EDIII; **Figure 1**).

EDII_FP_ contains multiple overlapping immunodominant B cell epitopes inducing broadly cross-reactive, weakly or non-neutralizing antibodies associated with antibody-enhanced severe DENV disease in both mice and humans (Stiasny et al., 2006; Lai et al., 2008; Crill et al., 2009; Balsitis et al., 2010; Beltramello et al., 2010; Zellweger et al., 2010). EDIII contains two well-characterized overlapping antigenic regions, one stimulating DENV complex cross-reactive antibodies varying in their neutralizing capabilities (Roehrig et al., 1998; Sukupolvi-Petty et al., 2007; Gromowski et al., 2008; Cockburn et al., 2012) and the other stimulating DENV serotype-specific, potently neutralizing antibodies associated with DENV serotype-specific immunity (Gromowski and Barrett, 2007; Sukupolvi-Petty et al., 2007; Crill et al., 2009; Beltramello et al., 2010). We examined multiple substitutions at EDII_FP_ residues G106 and L107 and at K310, E311, and P364 in the cross-reactive antigenic region of EDIII (EDIII_CR_; **Figure 1**). Final individual substitutions at these five residues were selected based upon their influence on *in vitro* VLP secretion and their effect on the reactivities of a panel of MAbs (**Table 2**).

EDII_FP_ substitutions tended to increase VLP secretion and knocked-out the reactivity of flavivirus group and sub-group cross-reactive MAbs. EDIII_CR_ substitutions tended to reduce VLP secretion relative to WT and ablated the reactivity predominately of DENV sub-complex cross-reactive MAbs. EDIII_CR_ substitutions were specifically selected not to interfere with the binding of potently neutralizing EDIII lateral ridge serotype-specific MAbs. Although human MAbs recognizing similar epitopes have been identified, a growing body of evidence suggests they may not comprise the main antigenic targets of the human neutralizing antibody response (Beltramello et al., 2010; Dejnirattisai et al., 2010; de Alwis et al., 2011; Wahala et al., 2012). The final pVD1-CRR plasmid, containing substitutions at all five of the sites across both EDII_FP_ and EDIII_CR_ antigenic regions (**Figure 1**), knocked-out or reduced the reactivity to below detectable levels of 11 cross-reactive monoclonal antibodies in our panel (**Table 2**). The cross-reactive MAbs whose reactivities were not significantly reduced were 1B7, a sub-group cross-reactive MAb that neutralizes all four DENV serotypes, and 10-D35A and D3-5C9-1, weakly (for DENV-2 only) and non-neutralizing DENV complex cross-reactive MAbs respectively.

### PROTECTIVE EFFICACY

#### Both pVD1-WT and pVD1-CRR vaccines induce similarly high titer IgG and protect mice from lethal DENV-1 challenge

**FIGURE 2:**
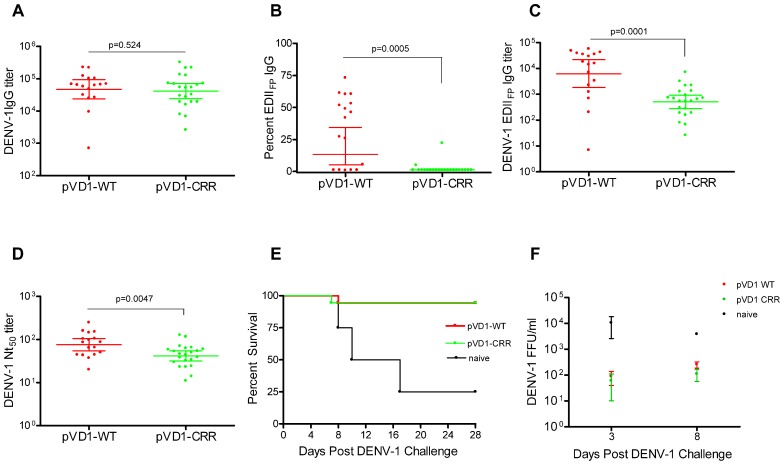
**Protective Efficacy: Both pVD1-WT and pVD1-CRR vaccines protect AG129 mice against lethal DENV-1 challenge. (A–D)** 12 week post vaccination immunogenicity of AG129 mice immunized (i.m.) with 100 μg of pVD1-WT or pVD1-CRR vaccines at 0, 4, and 8 weeks. Geometric means and 95% confidence intervals are depicted unless otherwise noted. All endpoint titers were log_10_ transformed and statistical significance was determined using the Mann–Whitney *U* test to account for non-normality of some transformed data. Panels B and C both used a one-tailed test since there was an *a priori* expectation that pVD1-CRR immunized mice would exhibit reduced EDII_FP_ recognizing IgG. **(A)** DENV-1 total IgG endpoint titers 12 weeks post immunization. **(B)** Percent of total DENV-1 IgG recognizing immunodominant EDII_FP_ epitopes, arithmetic means and 95% CI depicted. **(C)** Calculated DENV-1 EDII_FP_ IgG endpoint titers. **(D)** DENV-1 50% antigen focus-reduction micro neutralization titers (FRμNT_50_). A t test was utilized here as both data sets were normally distributed. **(E)** pVD1-WT and pVD1-CRR immunized AG129 mice were challenged with 1.1x10^5^ FFU of DENV-1 (Mochizuki strain, i.p.) at 12 weeks. Kaplan–Meier survival curves are shown. Both pVD1-WT (*n* = 18) and pVD1-CRR (*n* = 18) vaccinated animals had the same survival (94%), which was highly significant in comparison to naїve mice (*n* = 4, *p* = 0.0003). **(F)** Four mice from each vaccine treatment (not included in the survival curves) were *a priori* scheduled to be euthanized 3 and 8 DPC. DENV-1 titers (FFU/mL) were log_10_ transformed and analyzed using two-way ANOVA; error bars represent standard error of the mean (SEM). Vaccine treatment was highly significant (*p* < 0.0001). Bonferroni *post hoc* tests indicated that viremia of each vaccinated group was significantly lower than for naїve mice (*n* = 2) 3 DPC (*p* < 0.001), only pVD1-CRR vaccinated mouse viremia was significantly lower than naїve mice (*n* = 2) 8 DPC (*p* < 0.01) and there was no difference between WT or CRR vaccinated mouse viremia 3 or 8 DPC.

To test for vaccine protective efficacy we compared WT and CRR DENV-1 DNA vaccines by immunizing groups of AG129 mice (*n* = 26) and subsequently challenging with a lethal dose of homologous DENV-1 (Mochizuki strain). AG129 type I/type II IFN receptor knock-out mice have impaired neutralizing antibody responses (Schijns et al., 1994) and as expected our DNA vaccines were not as immunogenic in these mice as in immunocompetent mouse strains (data not shown). We therefore altered our standard immunization schedule from a single 100 μg boost 4 weeks following primary vaccination (100 μg) to two 100 μg boosts at 4 and 8 weeks. This schedule elicited IgG immune responses approaching the magnitude of those previously observed in other mouse strains with the two dose schedule (Chang et al., 2003; Hughes et al., 2012a,b). Mice were challenged at 12 weeks. Prior to challenge, mouse sera was collected to measure vaccine-induced humoral immune responses. We used ES-ELISA (Crill et al., 2009) to determine total DENV-1 IgG and DENV-1 EDII_FP_ IgG. Total DENV-1 IgG endpoint titers were similar between the two vaccines and averaged 7.7 × 10^4^ ± 1.5 × 10^4^ and 7.5 × 10^4^ ± 1.8 × 10^4^ for pVD1-WT and pVD1-CRR immunized mice respectively (*p* = 0.524; **Figure 2A**). However, pVD1-CRR immunized mice had significantly lower proportions of EDII_FP_ IgG than did pVD1-WT immunized mice, averaging 2.1 ± 0.96 and 33 ± 6.4% of the total IgG response respectively (*p* = 0.0005, **Figure 2B**). Only two pVD1-CRR immunized mice had measurable EDII_FP_ IgG (4.8 and 22%) with the remaining mice being below detectable levels (conservatively set to 1.0% for statistical analyses and EDII_FP_ IgG titer calculations). The proportion of EDII_FP_ IgG for WT immunized mice was large and variable, ranging from 0.8 to 73%, similar to that observed in DENV infected humans (Stiasny et al., 2006; Crill et al., 2009; Beltramello et al., 2010). Calculated EDII_FP_ IgG endpoint titers averaged 2.0 × 10^4^ ± 4.6 × 10^3^ and 1.1 × 10^3^ ± 3.4 × 10^2^ for WT and CRR immunized mice respectively (*p* = 0.0001, **Figure 2C**). Fifty percent neutralization (Nt_50_) titers, measured by FRμNT, averaged 91.1 and 48.8 for pVD1-WT and pVD1-CRR vaccinated mice respectively (*p* = 0.0047; **Figure 2D**). The lower Nt_50_ titer for pVD1-CRR immunized mice was likely due to reduced induction of EDIII_CR_ antibodies recognizing epitopes similar to those of neutralizing MAbs 1A1D-2 and 9D12 that lost all measurable reactivity for the pVD1-CRR tissue culture derived VLPs (**Table 1**). These results suggest that targeted substitution within EDII_FP_ can reduce the immunodominance of this region.

Age-matched naїve (18-week-old, *n* = 8), pVD1-WT and pVD1-CRR (*n* = 26 each) vaccinated animals were challenged with 1.1 × 10^5^ FFU of the mouse-brain adapted DENV-1 Mochizuki strain. This dose was >100 LD_5__0_ for 6–8-week-old mice (data not shown). All immunized mice except a single animal from each vaccine treatment were protected and survived challenge (94% survival) which was highly significant in comparison to naїve mice (25% survival, *p* = 0.0003; **Figure 2E**). Time to death of naїve mice ranged from 8 to 17 DPC and averaged 11.7 DPC; the single vaccinated animals died 7 and 8 DPC for WT and CRR vaccine treatments respectively. Surviving mice showed no signs of sickness. Although DENV-1 Mochizuki is a mouse-brain adapted virus, we observed limited neurological symptoms such as paralysis in terminally sick mice and most exhibited hunched and ruffled posture, lethargy, and a lack of interest in food and water leading to weight loss prior to being euthanized. Four mice from each vaccine treatment and two naїve mice were scheduled *a priori* to be euthanized at 3 DPC, a time point prior to any outward sign of disease, and 8 DPC, a few days after the initial signs of morbidity and mortality. Vaccinated mice exhibited 100-fold and 10-fold lower viremia 3 and 8 DPC respectively, compared to naїve controls; yet there was no difference in mean viremic titers between WT or CRR vaccinated mice at either time point (**Figure 2F**). A two-way ANOVA found vaccine treatment to be highly significant (*p* < 0.0001) and to account for 68% of the variation. Bonferroni *post hoc* tests indicated that viremia of each vaccinated group was significantly lower than for naїve mice 3 DPC (*p* < 0.001) whereas only pVD1-CRR immunized mouse viremia was significantly lower than naїve mice 8 DPC (*p* < 0.01).

Because DENV-1 Mochizuki strain is a mouse-brain adapted virus, we also determined virus titers of mouse-brain homogenates. Brains from both naїve and vaccinated mice were all negative 3 DPC (<50 FFU/g brain tissue). By 8 DPC, the DENV-1 titer of naїve mouse-brain tissue was 100–1000 times greater than for vaccinated mice. Of note, we only had tissue from a single 8 DPC naїve mouse-brain precluding rigorous statistical analysis. Nevertheless, the single 8 DPC naїve mouse-brain virus titer was 2.3 × 10^4^ FFU/g, geometric mean titers of WT and CRR vaccinated mice (*n* = 4 each) were 201 and 731 FFU/g of brain tissue respectively with three WT and two CRR vaccinated titers below the limits of assay detection (<50 FFU/g tissue). There was no difference in titer between WT and CRR vaccinated mouse-brains as determined by *t*-test or Mann–Whitney *U* test (data not shown). A Wilcoxon signed-rank test comparing all eight vaccinated mouse-brain titers to the 2.3 × 10^4^ titer for the naїve mouse-brain tissue strongly rejected the null hypothesis of no difference (*p* = 0.0059), suggesting that there was less virus in vaccinated compared to naїve mouse brains 8 DPC.

Consistent with an anamnestic response to DENV-1 challenge, both WT and CRR immunized surviving mice exhibited similar order of magnitude increases in Nt_50_ titer 28 DPC (GMT = 690, 95% CI 504–944 and =362, 95% CI 218–601 for WT and CRR respectively). Together, these data suggest that pVD1-WT and pVD1-CRR vaccines are similarly immunogenic and able to induce protective immunity against lethal homologous DENV-1 challenge in the AG129 DENV vaccine model.

### VACCINE SAFETY: CROSS-REACTIVE REDUCED VACCINE REDIRECTS IMMUNITY FROM IMMUNODOMINANT PATHOLOGICAL TOWARD PROTECTIVE RESPONSES

Groups of AG129 mice (*n* = 26) were immunized with a single 100 μg dose of pVD1-WT or pVD1-CRR vaccine and subsequently challenged with a sub-lethal dose of heterologous DENV-2 S221 12 weeks post vaccination, the same timeline used for the homologous DENV-1 protective efficacy challenge above. DENV-2 S221 is a mouse adapted isolate of Taiwanese strain PLO46 (Shresta et al., 2006) capable of producing lethal hemorrhagic disease in AG129 mice – similar to that observed in human DHF – via ADE when sub-protective levels of DENV immune sera are passively transferred to mice subsequently challenged with non-lethal doses of DENV-2 S221 (Balsitis et al., 2010; Zellweger et al., 2010). We hypothesized that pVD1-WT immunized mice would be susceptible to antibody-enhanced DENV disease due to high levels of vaccine-induced cross-reactive antibodies and that upon heterologous challenge; antibodies recognizing these epitopes would be stimulated anamnestically. Conversely, pVD1-CRR vaccinated mice should lack such cross-reactive antibody priming and secondary responses, be less susceptible to antibody enhanced viral replication, and exhibit increased survival.

#### DENV-1 CRR vaccinated mice have reduced levels of immunodominant EDII_FP_ IgG and reduced dengue disease mortality compared to DENV-1 WT vaccinated mice following DENV-2 challenge

**FIGURE 3:**
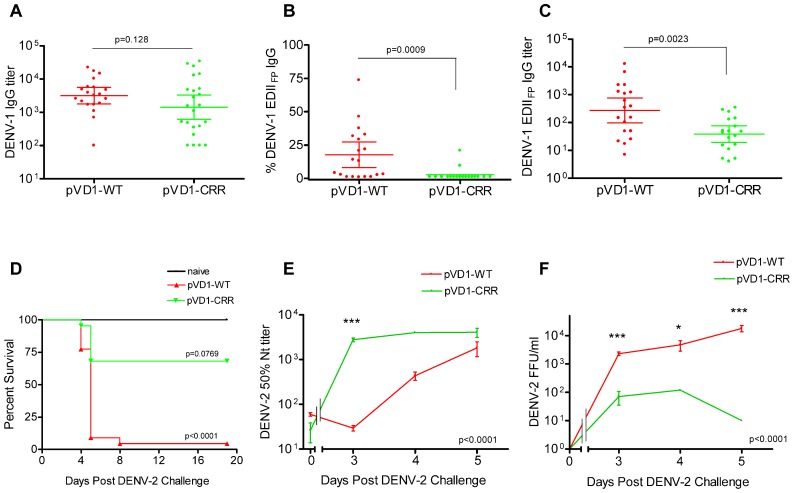
**DENV-1 CRR vaccine stimulates reduced levels of immunodominant cross-reactive EDII_FP_ IgG and reduces enhanced DENV-2 mortality. (A–C)** 12 week post vaccination immunogenicity of AG129 mice immunized (i.m.) with 100 μg of pVD1-WT or pVD1-CRR vaccines. Geometric means and 95% confidence intervals are depicted. Endpoint titers were log_10_ transformed and statistical significance was determined using the two-tailed Mann–Whitney *U* test. Panels B and C both used a one-tailed test since there was an *a priori* expectation that pVD1-CRR immunized mice would exhibit reduced EDII_FP_ recognizing IgG. **(A)** DENV-1 total IgG endpoint titers 12 weeks post immunization. **(B)** Percent of total DENV-1 IgG recognizing immunodominant EDII_FP_ epitopes, arithmetic means and 95% CI depicted. **(C)** Calculated DENV-1 EDII_FP_ IgG endpoint titers. **(D)** Survival of pVD1-WT and pVD1-CRR immunized AG129 mice following sub-lethal heterologous DENV-2 challenge. 12 weeks following vaccination, immunized mice (*n* = 22) or age matched naїve controls (*n* = 8) were challenged (i.p.) with 4.2 × 10^5^ FFU of DENV-2 S221. Kaplan-Meier survival curves and p values are shown. All naїve mice survived virus challenge with minimal signs of morbidity, pVD1-WT vaccinated mice suffered 95% mortality from enhanced DENV-2 disease (*p* < 0.0001, compared to naive). pVD1-CRR immunized mice exhibited 68% survival which did not differ from naїve mouse survival (100%, *p* = 0.0769) yet was significantly greater than pVD1-WT immunized mouse survival (4.5%, *p* < 0.0001). The Bonferroni multiple comparison adjusted α = 0.017. **(E)** pVD1-CRR vaccinated mice exhibited a rapid, large magnitude increase in DENV-2 neutralizing antibody titers following DENV-2 challenge. FRμNT_50_ titers determined with DENV-2 16681 virus on Vero cells, log_10_ transformed and the transformed data analyzed by two-way ANOVA (*p* < 0.0001). Bonferroni *post hoc* test significance is depicted with asterisks, error bars represent SEM. 0, 3, 4, and 5 DPC *n* = 4, 4, 5, and 9 for WT and 4, 4, 1, and 3 for CRR immunized mice respectively. **(F)** Viremia of pVD1-WT vaccinated mice increased rapidly 3–5 days after DENV-2 S221 challenge, whereas pVD1-CRR vaccinated mice had 30-fold, 40-fold, and at least 1800-fold lower viremia 3, 4, and 5 DPC (all CRR mice <10 FFU/mL) than did WT immunized mice. Virus titers were log_10_ transformed and the transformed data analyzed by two-way ANOVA (*p* < 0.0001). Bonferroni *post hoc* test significance is depicted with asterisks, error bars represent SEM. 3, 4, and 5 DPC *n* = 4, 5, and 6 for WT and 4, 1, and 3 for CRR immunized mice respectively.

To assess if pVD1-WT and -CRR vaccines induce different antibody repertoires in vaccinated mice we collected 12-week post-vaccination sera and utilized ES-ELISA (Crill et al., 2009) to determine vaccine induced DENV-1 IgG titers and the proportions and titers of IgG recognizing EDII_FP_ epitopes (**Figure 1**). There was no difference in total DENV-1 IgG GMT between pVD1-WT (3168, 95% CI 1792–5600; *n* = 20) and pVD1-CRR immunized mice (1429, 95% CI 621–3289, *n* = 23; *p* = 0.128, two-tailed Mann–Whitney *U* test; **Figure 3A**). The percent of total DENV-1 IgG recognizing EDII_FP_ epitopes averaged 17.7 ± 4.6% and 2.56 ± 1.2% for pVD1-WT and pVD1-CRR immunized mice respectively (*p* = 0.0009). Again, only two CRR immunized mice had detectable levels of EDII_FP_ IgG (9.4% and 21%) with all remaining mice having no measurable EDII_FP_ IgG (=1.0% for statistical analyses; **Figure 3B**). The calculated DENV-1 IgG GMT recognizing EDII_FP_ epitopes was significantly lower for CRR (38.6, 95% CI 19.4–76.9) than for WT immunized mice (272, 95% CI 97.2–760, *p* = 0.0023, one-tailed Mann–Whitney *U* test; **Figure 3C**). These results again suggest that pVD1-WT and pVD1-CRR vaccines are similarly immunogenic overall, but that the pVD1-CRR vaccine induces less IgG recognizing immunodominant EDII_FP_ epitopes.

Age-matched naїve (*n* = 10), pVD1-WT, and pVD1-CRR (*n* = 26 each) immunized mice were challenged (i.p.) with a sub-lethal dose (4.2 × 10^5^ FFU) of DENV-2 S221 12 weeks following immunization for vaccine treatment groups. All naїve mice survived DENV-2 challenge, though they did exhibit signs of morbidity including decreased activity, coat ruffling, and weight loss. pVD1-WT vaccinated mice suffered from severely enhanced DENV-2 pathology, including hemorrhagic manifestations producing 95% mortality (*p* < 0.0001 compared to naїve). Conversely, pVD1-CRR vaccinated mice exhibited reduced DENV-2 disease enhancement and 68% survival which did not differ from naїve mouse survival (100%, *p* = 0.0769) and yet was much greater than WT vaccinated mouse survival (4.5%, *p* < 0.0001, the Bonferroni multiple comparison adjusted α = 0.017; **Figure 3D**). Sick mice began exhibiting external signs of morbidity late on day 3 and reached terminal endpoints 4.5–5.5 DPC for both groups with one WT vaccinated mouse surviving to 8 DPC.

To characterize which humoral immune determinates might be responsible for the reduced disease severity and mortality of CRR vaccinated mice we examined sera from 3, 4, and 5 DPC. Four animals in each vaccine treatment were selected *a priori* for sacrifice 3 DPC, a time point when mice exhibited no outward signs of morbidity. Four and 5 DPC sera were obtained from terminally sick mice via cardiac puncture just prior to euthanasia. Thus, sample sizes were limited on some of these days for pVD1-CRR vaccinated mice since there was limited mortality relative to pVD1-WT vaccinated mice. Serum specimens were collected and DENV-2 Nt_50_, viremia, and ES-ELISA titers were determined. DENV-2 neutralization was strongly correlated with survival; pVD1-CRR immunized mice exhibited a rapid, large magnitude rise in DENV-2 neutralization whereas in pVD1-WT immunized mice, neutralization was lower and increased more slowly (*p* < 0.0001 in a two-way ANOVA, **Figure 3E**). Three DPC Nt_50_ titers of pVD1-CRR vaccinated mice were nearly 100 times greater than those of pVD1-WT vaccinated mice (CRR = 2783.6, WT = 29.5; Bonferroni *post hoc* test *p* < 0.001). This was a >100-fold increase in DENV-2 neutralization compared to pre-challenge for CRR vaccinated mice whereas it was a twofold decrease over the same time period for WT vaccinated mice. Nt_50_ titers of CRR vaccinated mice remained high 4 and 5 DPC whereas those of WT vaccinated mice slowly increased and remained twofold lower than CRR vaccinated mice by 5 DPC. Viremia was negatively correlated with DENV-2 neutralization and positively correlated with mortality (**Figure 3F**). 3 DPC mean viremia of WT vaccinated mice was 33 times higher (2325 FFU/mL) than that of CRR vaccinated mice (70 FFU/mL; Bonferroni *post hoc* test *p* < 0.001) with two of four CRR immunized mouse sera below the limits of assay detection (10 FFU/mL). WT viremia continued to increase 4–5 DPC and by 5 DPC was at least 1800 times higher than for CRR immunized mice where all individuals had dropped to below detectable levels (ave = 1.8 × 10^4^ and <10 FFU/mL for WT and CRR immunized mice respectively; *p* < 0.001 with Bonferroni *post hoc* test in two-way ANOVA). These findings imply that even though some CRR vaccinated mice were terminally ill 5 DPC, they had cleared their viremia, whereas WT vaccinated mice still exhibited increasing viremia during this time of maximal mortality. Thus, the rapid, large magnitude increase in DENV-2 neutralization of pVD1-CRR vaccinated mice was consistent with an anamnestic response and/or potentially redirected immunity, whereas the slow rise in DENV-2 neutralization of WT vaccinated mice was more characteristic of a primary immune response.

**FIGURE 4:**
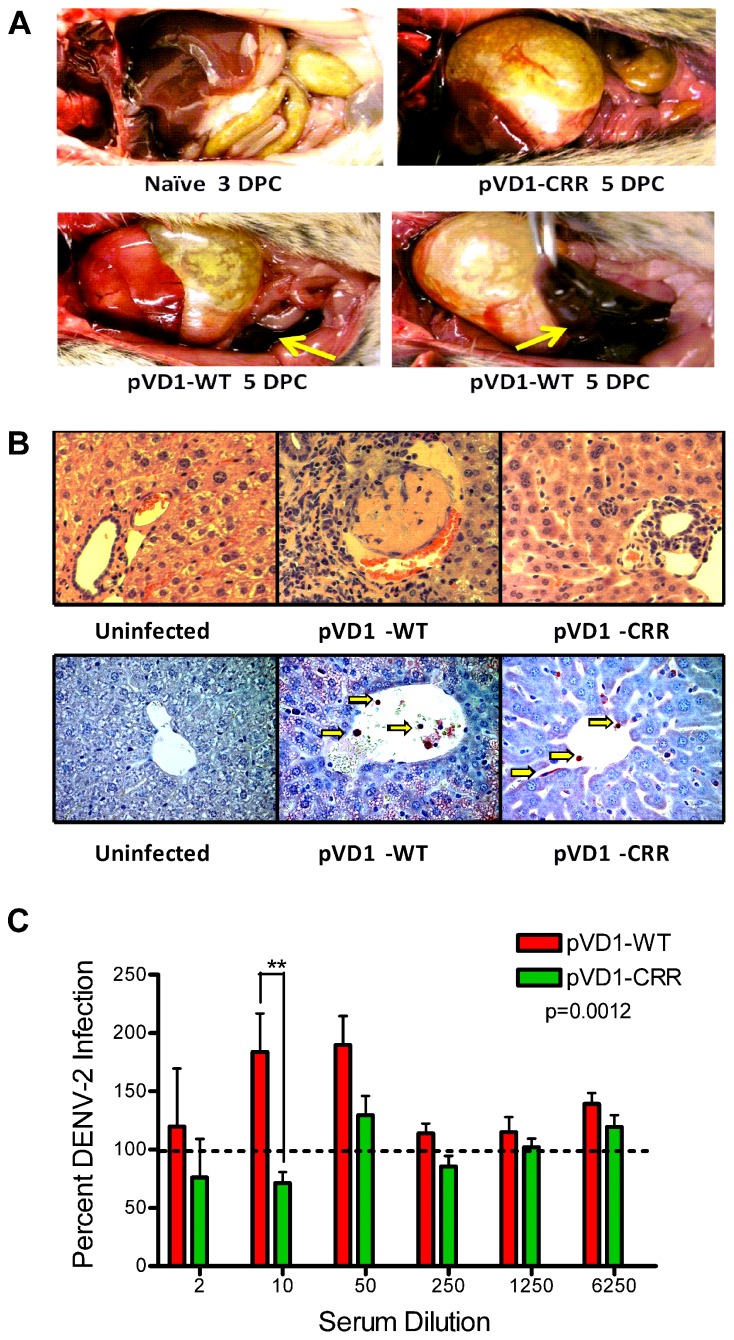
**Pathophysiology of enhanced DENV disease in vaccinated AG129 mice. (A)** Enhanced DENV disease associated vascular leak pathology of pVD1-WT and -CRR vaccinated mice following sub-lethal heterologous DENV-2 infection. No pathology was visible in naїve or vaccinated mice 3 DPC, yet by 4 and 5 DPC mice succumbing to enhanced disease exhibited severe vascular leakage associated pathology. The same mouse is shown in the bottom two photos to highlight the severe intestinal hemorrhage observed in pVD1-WT but not in pVD1-CRR vaccinated mice (arrows). **(B)** Histology of uninfected, pVD1-WT, and pVD1-CRR vaccinated mouse livers 3 DPC. The top row is hematoxylin and eosin stained liver sections and the bottom row is immunohistochemistry for DENV-2 NS1 protein of the same individual animals. NS1+ mononuclear inflammatory cells and sinusoidal endothelial cells stain red in their cytoplasm and are visible (arrows) in liver tissue from vaccinated mice but not uninfected mice. Multiple sections from multiple animals were examined and single representatives are shown. All photos were taken at 400x magnification. **(C)** *In vitro* DENV-2 enhancement by pVD1-WT and pVD1-CRR vaccinated serum 12 weeks following vaccinations and one day prior to DENV-2 challenge. Consistent with the *In vivo* results, pVD1-WT vaccinated serum significantly enhanced DENV-2 infection whereas pVD1-CRR vaccinated serum did not (*p* = 0.0012). The data are representative of two independent experiments of 4 pools of 6–7 individual serum specimens for each vaccine treatment and were analyzed with two-way ANOVA and Bonferroni *post hoc* test significance at individual dilutions is depicted with asterisks.

pVD1-WT vaccinated AG129 mice suffering from lethally enhanced DENV-2 disease exhibited pathology consistent with ADE disease described in passive transfer studies with this mouse model (Balsitis et al., 2010; Zellweger et al., 2010). 3 DPC neither vaccinated nor naїve mice exhibited visible pathological symptoms upon necropsy. All terminally ill mice however had pale, blood-depleted livers, increased intestinal capillary blood flow, and enlarged gaseous stomachs, symptomatic of fluid accumulation caused by vascular leakage. Only WT vaccinated mice exhibited the most severe gastrointestinal hemorrhage (**Figure 4A**). Although gross pathology upon necropsy of vaccinated mice was not observed until 4 DPC, H&E staining of liver tissue revealed hepatitis pathology 3 DPC with increasing severity 4 and 5 DPC. Consistent with the reduced gross morbidity and mortality of pVD1-CRR compared to pVD1-WT vaccinated mice, CRR vaccinated mice reaching terminal endpoints exhibited mild lymphoplasmacytic portal, multifocal suppurative, and necrotizing hepatitis, including vacuolar vein congestion. However, pVD1-WT vaccinated mice exhibited moderate to severe lymphoplasmacytic, necrotizing, and multifocal portal hepatitis and in some individuals extensive vascular thrombosis and advanced vacuolar degeneration (**Figure 4B**). Severe DENV pathology via ADE posits that there should be increased infection of FcγR bearing monocytic cells in humans (Morens, 1994) and in AG129 mice, previous studies found that antibody-enhanced mortality was associated with increased DENV infection and replication in liver sinusoidal endothelial cells (Balsitis et al., 2010; Zellweger et al., 2010). To assess viral replication in liver tissue we used immunostaining for DENV-2 NS1 protein which is expressed during viral replication. Of note, although we did not attempt to quantify differences between WT and CRR vaccine treatments, mononuclear inflammatory cells in the portal areas and sinusoidal endothelial cells of both WT- and CRR vaccinated liver tissue were NS1 positive by 3 DPC, suggestive of active viral replication in FcγR bearing cells of the liver, and liver tissue of naїve mice was negative for NS1 by immunostaining (**Figure 4B**). Lastly, pre-challenge pVD1-WT vaccinated mouse serum (1 day prior to DENV-2 challenge) significantly enhanced DENV-2 replication in FcγR bearing human K562 cells, and pVD1-CRR immunized mouse sera did not (**Figure 4C**). In summary, the high levels of DENV-1 vaccine induced cross-reactive EDII_FP_ IgG prior to challenge, increased pathology and mortality, higher viremia, and active viral replication in FcγR bearing cells of WT immunized mice are all consistent with previous descriptions of antibody-enhanced DENV disease in AG129 mice (Balsitis et al., 2010; Zellweger et al., 2010).

#### Cross-reactivity reduced DNA vaccination sculpts immune memory so that response to virus infection is redirected: reducing pathological and increasing protective immunity

**FIGURE 5:**
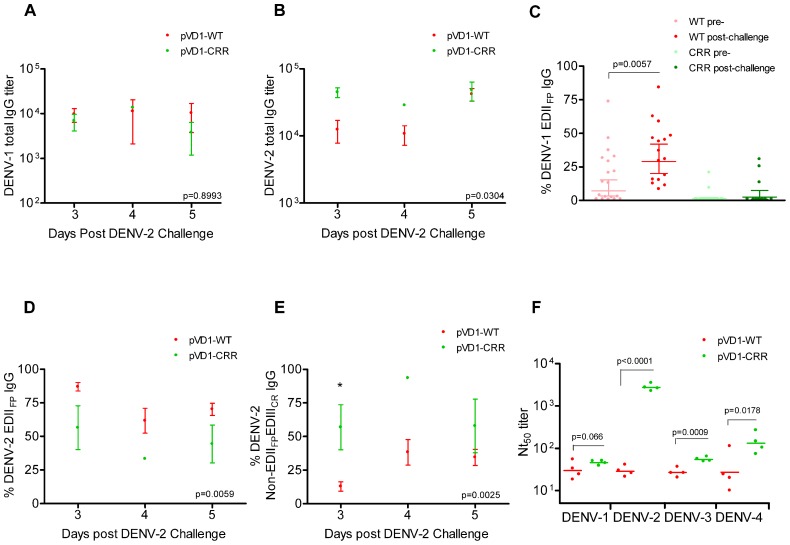
**DENV-1 CRR vaccination sculpts immunity to redirect secondary immunity to heterologous dengue infection.** AG129 mice were immunized (i.m.) with 100 μg of pVD1-WT or pVD1-CRR vaccines and challenged at 12 weeks with heterologous DENV-2 S221 (4.2x10^5^ FFU, i.p.). Arithmetic means and SEM are depicted in A, B, D, and E and analyzed with two-way ANOVA. Bonferroni *post hoc* test significance is depicted with asterisks. **(A)** DENV-1 total IgG endpoint titers of pVD1-WT and -CRR immunized mice 3, 4, and 5 DPC with DENV-2. Three, 4, and 5 DPC *n* = 4, 3, and 8 and 4, 1, and 3 for WT and CRR vaccines respectively. **(B)** DENV-2 total IgG endpoint titers of pVD1-WT and -CRR vaccinated mice. **(C)** Percent DENV-1 epitope-specific responses pre- and post-challenge for IgG recognizing immunodominant cross-reactive EDII_FP_ epitopes. pVD1-WT immunized mice had larger populations of EDII_FP_ post DENV-2 challenge than did pVD1-CRR immunized mice (ave = 35.5 and 7.65%, respectively, *p* = 0.0004). pVD1-WT immunized mice also exhibited an increase in EDII_FP_ IgG from pre- to post-DENV-2 challenge (ave = 17.7 and 35.5%, respectively, *p* = 0.0057. Geometric means and 95% CI are depicted. Statistical significance was determined with a one-tailed Mann–Whitney *U*. The Bonferroni α = 0.025. **(D,E)** DENV-2 epitope specific IgG responses post DENV-2 challenge of pVD1-WT and pVD1-CRR immunized AG129 mice; 3, 4, and 5 DPC *n* = 4, 5, and 9 and 4, 1, and 2 for WT and CRR immunized mice respectively. (**D)** Percent DENV-2 IgG recognizing immunodominant cross-reactive EDII_FP_ epitopes. **(E)** Percent DENV-2 IgG recognizing E protein epitopes outside of immunodominant EDII_FP_ and EDIII_CR_ antigenic regions (Non-EDII_FP_EDIII_CR_). **(F) **FRμNT_50_ titers of pVD1-WT and -CRR immunized mouse sera 3 DPC with DENV-2 against DENV-1, DENV-2, DENV-3, and DENV-4. Bars represent GMT, statistical significance was determined with a one-tailed *t*-test. All titers were log_10_ transformed prior to analysis.

We used DENV-1 and DENV-2 ES-ELISA to quantify and differentiate humoral immune responses to DENV-1 vaccination from DENV-2 challenge. Supporting the hypothesis that WT vaccinated mice were slower to mount DENV-2 specific immunity due to DENV-1 primed cross-reactive memory responses, WT vaccinated mice had lower DENV-2 total IgG titers yet larger populations of both DENV-1 and DENV-2 EDII_FP_ IgG than did CRR vaccinated mice following heterologous challenge (**Figure 5**). Total DENV-1 IgG titers did not differ between vaccine treatments 3, 4, and 5 DPC with DENV-2 (**Figure 5A**); however, DENV-2 IgG titers were higher for pVD1-CRR than for pVD1-WT immunized mice (**Figure 5B**). Nevertheless, pVD1-WT vaccinated mice had significantly larger populations of EDII_FP_ IgG for both DENV-1 (**Figure 5C**) and DENV-2 (**Figure 5D**) than did pVD1-CRR vaccinated mice. Not only did pVD1-WT mice have larger populations of cross-reactive EDII_FP_ IgG following heterologous challenge, there was also a significant increase in DENV-1 EDII_FP_ IgG post challenge when compared to the 12 week pre-challenge data (**Figure 5C**). DENV-1 EDII_FP_ IgG GMT of WT immunized mice increased from 17.7 ± 4.6% pre-challenge to 35.5 ± 5.4% of the total IgG response post DENV-2 challenge. However, there was no increase in DENV-1 EDII_FP_ IgG for CRR immunized mice before and after challenge (*p* = 0.364, Mann–Whitney *U*). We also analyzed individual pairs of pre- and post-DENV-2 challenge sera from WT immunized mice for DENV-1 EDII_FP_ IgG and this analysis also demonstrated a significant increase post-challenge (*p* = 0.0138) and a significant effect of individual serum pairing (*p* = 0.0267, one-tailed paired *t*-test; data not shown). Together, these results suggest a strong memory response to cross-reactive E protein epitopes in DENV-2 that were primed by pVD1-WT vaccination and that such an immunodominant anamnestic response was lacking in pVD1-CRR immunized mice.

ES-ELISA can also determine the proportion of IgG recognizing E epitopes outside of the manipulated EDII_FP_ and EDIII_CR_ antigenic regions in the pVD1-CRR vaccine (non-EDII_FP_EDIII_CR_ IgG; **Figure 1**;Hughes et al., 2012a). We hypothesized that pVD1-CRR immunized mice, with their reduced EDII_FP_ response, should exhibit increased capacity to redirect humoral immune responses to increase the DENV-2 non-EDII_FP_EDIII_CR_ antibody populations concomitant with their reduced EDII_FP_ response and that WT immunized mice should show the opposite pattern. Supporting this prediction, CRR vaccinated mice had significantly larger populations of DENV-2 non-EDII_FP_EDIII_CR_ IgG 3, 4, and 5 DPC with DENV-2 than did pVD1-WT immunized mice (*p* = 0.0025 in two-way ANOVA; **Figure 5E**). This was the same time period that WT immunized mice exhibited maximum enhanced DENV-2 disease mortality (**Figure 3D**) and significant increases in EDII_FP_ IgG (**Figures 5C,D**); whereas CRR immunized mice exhibited 100-fold increased DENV-2 neutralization (**Figure 3E**). Moreover, a comparison of DENV-1 non-EDII_FP_EDIII_CR_ IgG for individual WT immunized mice pre- and post-challenge showed a significant decrease (*p* = 0.0435) in this antibody population 3–5 DPC and a significant effect of individual mouse serum pairing on this decrease (*p* = 0.0111, one-tailed paired *t*-test; data not shown). A decrease in this DENV-1 antibody population of WT immunized mice is what one would expect if there is a trade-off between immunodominant secondary responses to EDII_FP_ and non-EDII_FP_EDIII_CR_ epitopes. Antibody stimulated from epitopes outside EDII_FP_ and EDIII_CR_ antigenic regions altered in our pVD1-CRR vaccine include neutralizing DENV complex reactive antibodies such as MAb 1B7 primed by either vaccine and boosted by DENV-2 challenge (**Table 2**) and/or DENV-2 serotype-specific neutralizing antibodies stimulated only by the challenge virus.

Three non-mutually exclusive mechanisms could explain the rapid increase in DENV-2 neutralizing antibody by CRR immunized mice 3 DPC: (1) anamnestically increased complex cross-reactive neutralizing antibody not altered by the substitutions introduced in the pVD1-CRR vaccine (e.g., 1B7-like), (2) primarily increased DENV-2-specific neutralizing antibody, or (3) increased relative neutralizing capability of CRR vaccinated sera by these or other neutralizing antibody populations due to a lack of steric interference by the large populations of EDII_FP_ IgG present in WT vaccinated mice (Ndifon et al., 2009). To test if the increased Nt_50_ titers of CRR vaccinated mice were due to increases in DENV complex cross-reactive neutralizing antibodies we determined the Nt_50_ titers for CRR and WT vaccinated mouse serum 3 DPC against DENV-1, DENV-3, and DENV-4 (**Figure 5F**). The mean (*n* = 4 each) Nt_50_ titers for CRR and WT vaccinated mice respectively were 46 and 33 for DENV-1 (*p* = 0.066), 55 and 27 for DENV-3 (*p* = 0.0009), and 147 and 42 for DENV-4 (*p* = 0.0178; one-tailed *t*-test for all). These 1.5-, 2.0-, and 3.5-fold higher DENV-1, -3, and -4 Nt_50_ titers for CRR vaccinated mice did not approach the nearly 100-fold greater DENV-2 Nt_50_ titer of CRR immunized mice 3 DPC (2785 and 29 respectively). These data suggest that CRR vaccinated mice appear to have increased complex cross-reactive neutralizing antibody relative to WT vaccinated mice; but this antibody class either neutralizes DENV-2 more efficiently than other DENV serotypes, or there are also increased proportions of DENV-2 specific neutralizing antibody in CRR vaccinated serum by 3 DPC with DENV-2.

**FIGURE 6:**
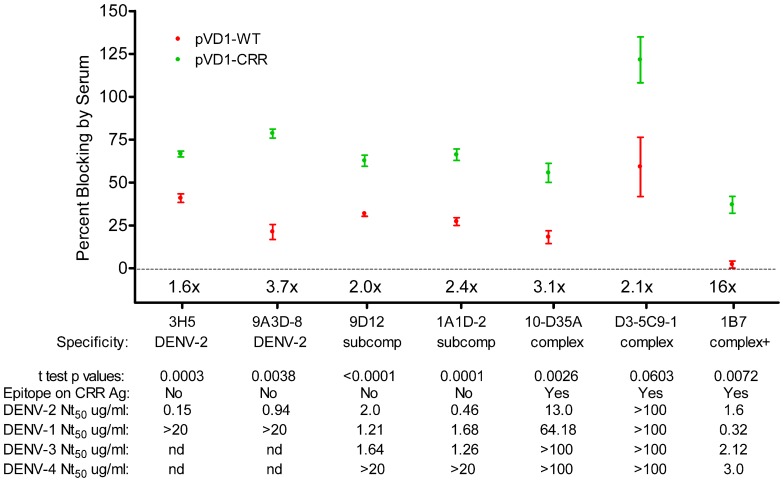
**DENV-1 CRR vaccination redirects immune responses to DENV-2 to increase a diversity of neutralizing antibodies.** Percent blocking of labeled monoclonal antibodies (MAbs) by pVD1-WT and pVD1-CRR immunized mice 3 DPC to DENV-2 as determined by blocking ELISA. AG129 mice were immunized (i.m.) with 100 μg of pVD1-WT or pVD1-CRR vaccines and challenged at 12 weeks with a sub-lethal dose of heterologous DENV-2 S221 (4.2 × 10^5^ FFU, i.p.). 3 DPC sera of the four mice from each vaccine treatment were pooled and percent blocking was determined independently four times in duplicate. To facilitate comparison between vaccine treatments across the different MAbs, x-fold increases in blocking by pVD1-CRR vaccinated sera relative to pVD1-WT vaccinated sera are depicted above the *x*-axis for each MAb. Below the MAbs, serotype specificity is depicted (DENV-2, DENV-2 type-specific; subcomp, DENV sub-complex cross-reactive; complex, DENV complex cross-reactive and complex + supercomplex cross-reactive). Below specificity, *p*-values from a two-tailed t test comparing percent blocking for each vaccinated sera are depicted. Below that the presence or absence of the epitope recognized by each MAb on the DENV-1 CRR VLP antigen is depicted (all MAbs recognize epitopes found in the DENV-2 challenge virus). For example, 3H5 and 9A3D-8 as DENV-2 specific antibodies are absent on both pVD1-WT and pVD1-CRR VLP antigens; 9D12 and 1A1D-2 reactivity are ablated by the EDIII_CR_ substitutions in the pVD1-CRR plasmid (**Table 2**) but present in the WT DENV-1 antigen; and 10-D35A, D3-5C9-1, and 1B7 reactivities were not altered by the substitutions introduced into in the pVD1-CRR plasmid and hence are present on both DENV-1 WT and CRR immunization antigens. FRμNT_50_ titers for each MAb against DENV-2, -1, -3, and -4 in μg/mL are also shown.

To further characterize the epitopes recognized by the antibodies responsible for the rapid rise in DENV-2 neutralization observed in CRR vaccinated mice, we tested the ability of 3 DPC sera to block the binding of labeled MAbs in a DENV-2 epitope-blocking ELISA. We examined the blocking by WT and CRR vaccinated mouse sera for MAbs recognizing complex cross-reactive epitopes present in both DENV-1 vaccines and in the DENV-2 challenge virus (1B7, 10-D35A, and D3-5C9-1), MAbs recognizing sub-complex cross-reactive epitopes knocked out in the CRR vaccine but present in the WT vaccine and DENV-2 challenge virus (1A1D-2 and 9D12), and MAbs recognizing DENV-2 serotype-specific epitopes present only in the challenge virus (9A3D-8 and 3H5). Surprisingly, pVD1-CRR vaccinated mouse sera exhibited significantly greater blocking than did WT vaccinated sera for all of these MAbs with the exception of D3-5C9-1 the only non-neutralizing MAb against DENV-2, implying that CRR immunized mice had higher titers of antibody recognizing the same or similar epitopes as these labeled MAbs (**Figure 6**). The greatest relative increase in blocking was for DENV complex, neutralizing MAb 1B7 which exhibited 16-fold greater blocking by CRR than the 2.25% blocking by WT (*p* = 0.0072, two-tailed *t*-test), implying greater 1B7-like neutralizing antibody in CRR vaccinated mouse sera than in WT mouse sera, and consistent with increased anamnestic induction of normally sub-dominant 1B7-like antibody in CRR immunized mice. CRR vaccinated mice also had 3.7 and 1.6-fold larger populations of DENV-2 serotype-specific potently neutralizing 3H5 and 9A3D-8 like antibodies than did WT vaccinated mice (*p* = 0.0003 and *p* = 0.0058, respectively), 2.4- and 2-fold greater sub-complex cross-reactive neutralizing 1A1D-2 and 9D12 like antibodies (*p* = 0.0001 and *p* < 0.0001 respectively), and 3.1-fold more DENV complex cross-reactive weakly neutralizing 10-D35A like antibodies (*p* = 0.0026). There was a non-significant 2.1-fold increase in complex cross-reactive MAb D3-5C9-1. We next determined the neutralizing capabilities of these seven MAbs since not all had been previously published in the literature (**Figure 6**). Not surprisingly, DENV-2 serotype-specific MAbs 3H5 and 9A3D-8 exhibited potent DENV-2 neutralization (Nt_50_ = 0.15 and 0.94 μg/mL respectively). 1A1D-2 also potently neutralized DENV-2 (0.46 μg/mL) although it neutralized DENV-1 and DENV-3 about threefold less than DENV-2, similar to the DENV-2 neutralizing capabilities of both 9D12 and 1B7. 10-D35A exhibited weak neutralization of DENV-2 (13.04 μg/mL), and D3-5C9-1 did not neutralize any DENV serotype. Together, these results begin to tease apart the individual humoral immune components responsible for the polyclonal response of vaccinated mice to heterologous infection. The 100-fold increase in DENV-2 neutralization appeared to be due to a combination of large increases in cross-reactive neutralizing antibodies, similar to 1B7, but also to relative increases and large populations of potently neutralizing serotype-specific and sub-complex cross-reactive antibodies. Thus, in response to heterologous DENV-2 infection, pVD1-CRR immunized mice exhibited rapid increases in neutralizing antibody populations with diverse patterns of reactivity that not only increased protection from enhanced DENV-2 disease mortality but also exhibited increased neutralization breadth across the DENV complex.

## DISCUSSION

In this report we constructed WT and CRR DENV-1 DNA vaccine candidates and compare their humoral immunogenicity as it relates to vaccine safety and efficacy using the AG129 mouse dengue disease model. The CRR vaccine was specifically engineered as a second generation vaccine candidate to reduce the potential for vaccine-induced susceptibility to severe dengue disease via ADE, a theoretical safety concern for dengue vaccine development (Miller, 2010; Murphy and Whitehead, 2011; Thomas, 2011; Heinz and Stiasny, 2012). CRR immunized mice had significantly reduced titers of EDII_FP_ antibodies, induced from cross-reactive immunodominant epitopes associated with ADE and increased pathology in humans and dengue animal models (Goncalvez et al., 2007; Balsitis et al., 2010; Beltramello et al., 2010; Dejnirattisai et al., 2010; Zellweger et al., 2010). We further demonstrate that by disrupting these epitopes we can redirect the immunodominance hierarchy away from pathogenic toward normally subdominant, potently neutralizing epitopes. WT vaccinated mice had high EDII_FP_ antibody titers and enhanced a heterologous, sub-lethal DENV-2 infection into a DHF-like disease pathology resulting in 95% mortality. CRR vaccinated mice however, lacking measurable EDII_FP_ antibodies, exhibited increased protective, neutralizing immunoglobulins, and had significantly reduced morbidity and mortality that did not differ from naive mice.

The location of E protein substitutions introduced into the pVD1-CRR vaccine construct used in this study was based upon previously published work in DENV-2 (Crill et al., 2009; Hughes et al., 2012a). We previously constructed DENV-2 CRR DNA vaccines with substitutions knocking out cross-reactive epitopes in the EDII_FP_, in EDIII, and in both EDII_FP_ and EDIII and compared their immunogenicity with each other and with the WT DENV-2 vaccine (Hughes et al., 2012a). All vaccines induced similar high-titer DENV-2 neutralizing antibody responses in Swiss Webster mice. In *in vitro* ADE assays, sera from WT immunized mice significantly enhanced DENV-1, -2, and -3 replication and sera from mice immunized with EDII_FP_ or EDIII substitutions enhanced DENV-1 or DENV-3, only serum from mice immunized with vaccines containing CRR substitutions in both EDII_FP_ and EDIII lacked enhancing capabilities for all serotypes at the lowest tested serum dilution (1:2). We observed that vaccines containing substitutions in EDIII exhibited a non-significant trend toward reduced DENV-2 neutralizing antibody titers but that the vaccine containing substitutions in both EDII_FP_ and EDIII also showed the greatest increase in heterologous neutralization. Based upon these observations we decided to incorporate substitutions in both EDII_FP_ and EDIII into the DENV-1 CRR vaccine construct.

Our results, suggestive of vaccine-induced severe dengue disease and mortality via ADE, are consistent with and supported by two recent studies of ADE-induced dengue disease in AG129 mice using passive transfer of DENV immune sera (Balsitis et al., 2010; Zellweger et al., 2010). These studies found that administering 10^4^, 10^5^, or 10^6^ pfu of DENV-2 S221 was sub-lethal to naїve AG129 mice receiving passively transferred normal mouse serum 24 h prior to challenge. However, mice receiving heterologous DENV-1, -3, or -4 immune sera 24 hr prior to challenge died 4–5 DPC from severe dengue disease resembling DHF. The disease pathology was similar to that seen in human DHF. Confirmation that this enhanced DHF-like disease and mortality resulted from ADE was supported by passive transfer of enhancing EDII_FP_ MAb 4G2 and by rescue from enhanced mortality by passive transfer of 4G2 F(ab′)2 fragment unable to bind FcγR (Balsitis et al., 2010). In this study, we found that when exposed to normally sub-lethal doses of DENV-2 S221, pVD1-WT vaccinated mice suffered 95% mortality, shared many of these same symptoms and that the high viremia and enhanced disease pathology and mortality were significantly reduced by immunization with a modified pVD1-CRR vaccine.

An important finding in this study was the rapid induction of diverse DENV-2 neutralizing antibodies by pVD1-CRR vaccinated mice in response to heterologous DENV-2 infection. There was a large increase in 1B7-like antibody for CRR vaccinated mice, supporting the importance of this class of neutralizing antibody. However, the majority of the increase in DENV-2 neutralization by CRR vaccinated mice appeared to be due to DENV-2 serotype-specific neutralizing antibody, since DENV-1, -3, and -4 neutralization only increased moderately. Neither pVD1-WT nor pVD1-CRR immunized mice were primed for DENV-2 serotype-specific neutralizing antibody responses, how then could this antibody class be so potently increased during early acute heterologous DENV-2 infection in CRR immunized mice? A recent study examining humoral and cellular immune response to primary DENV-1 and secondary DENV-2 infections in AG129 mice is relevant to this discussion (Zompi et al., 2012). These authors observed that by 6 days post-infection naїve B cells had differentiated into plasma cells capable of producing both serotype-specific and cross-reactive neutralizing antibody. Notably though, DENV-2-specific plasma cells peaked at 6 days post-primary DENV-2 infection, but in secondary DENV-2 infected mice this peak was delayed until 9 days post-infection. These findings were interpreted to suggest that secondarily infecting DENV-2 antigen was more readily captured by cross-reactive DENV-1 derived memory B cells and/or antibody, preventing the early binding and activation of naїve B cells during secondary DENV-2 infection. A similar phenomenon could explain the more rapid production of DENV-2 serotype-specific neutralizing antibody in pVD1-CRR immunized mice by 3 DPC with DENV-2. We believe such a process to be a major mechanistic explanation behind the immune redirection associated with the reduced DENV-2 disease enhancement and mortality observed in pVD1-CRR immunized mice.

A surprising finding was the rapid increase of sub-complex cross-reactive antibodies in pVD1-CRR immunized mice following DENV-2 infection, since antibodies recognizing epitopes similar to MAbs 1A1D-2 and 9D12 should have been primed by pVD1-WT but not by pVD1-CRR vaccination. One possible explanation is that the CRR substitutions introduced into EDIII did not completely knock-out these epitopes. This is possible because EDIII_CR_ antibodies recognize epitopes in a complex, conformational antigenic surface (Sukupolvi-Petty et al., 2007; Gromowski et al., 2008; Pierson et al., 2008; Cockburn et al., 2012). However, the increased production of sub-complex cross-reactive antibodies in CRR vs. WT vaccinated mice may simply be due to a reduced capability of WT vaccinated mice to produce this sub-dominant antibody class in response to DENV-2 infection (Zompi et al., 2012). This study did not address the possible role of antibody interference in explaining the rapid increases in DENV-2 neutralization observed in pVD1-CRR vaccinated mice (Ndifon et al., 2009). Weakly or non-neutralizing antibodies recognizing EDII_FP_ epitopes could sterically interfere with the binding of potently neutralizing antibodies recognizing either EDIII or recently identified inter-dimer quaternary epitopes (de Alwis et al., 2012). The lack of large populations of EDII_FP_ IgG in pVD1-CRR vaccinated mouse serum can therefore increase its relative neutralization and we suspect this phenomenon to also play a role in the increased neutralization of pVD1-CRR vaccinated mice.

Because this study utilized active vaccination, the increased survival and decreased disease incidence and severity of pVD1-CRR vaccinated mice could result from additional factors such as differences in cellular immunity. Even in IFNα/β, IFNγ receptor-deficient AG129 mice, cellular immunity could play an important role in either protection from or enhancement of dengue infection and disease (Yauch et al., 2009, 2010; Zompi et al., 2012). As a part of a recently published CD4+ T cell study we developed an overlapping peptide library for DENV-2 prM and E proteins. Mice immunized with DENV-2 CRR vaccines, containing substitutions at the same EDII_FP_ and EDIII_CR_ residues, did not differ in CD4+ or CD8+ T cell recognition for the WT DENV-2 peptides, implying that the substitutions introduced into DENV CRR vaccines are either not located in T cell epitopes or do not alter existing T cell epitopes in these regions (Hughes et al., 2012b; Hughes and Chang, unpublished data).

Most immunodominant DENV T cell epitopes have been localized to the non-structural proteins, particularly NS3, and the DNA vaccines utilized in this study do not contain the genes coding for non-structural proteins (Mathew and Rothman, 2008). T cell epitopes that have been localized to the prM/E structural proteins have predominately been localized to regions outside those modified in our CRR vaccine (Yauch et al., 2009; Duangchinda et al., 2010). Roehrig et al. (1994) found that a DENV-2 E protein peptide containing EDII_FP_ residues did not induce virus-reactive CD4+ T cells *in vitro* or prime H-2b CD4+ virus-specific T cells whereas peptides spanning EDIII residues 302–333 or 352–368 could weakly induce such priming. It is therefore possible that pVD1-CRR substitutions could increase CD4+ T cell priming beyond the low levels observed with the WT peptides to boost humoral immunity or that the EDII_FP_ and/or EDIII_CR_ substitutions could increase CD8+ T cell activation relative to WT vaccine; possibly accounting for the observed survival differences. However, similar pathologies were observed following passive transfer of DENV immune sera (Balsitis et al., 2010; Zellweger et al., 2010). In the present study, we observed *in vitro* DENV-2 enhancement with pVD1-WT vaccinated mouse sera that was not observed with pVD1-CRR vaccinated sera; and in our recently published study, enhanced disease mortality of AG129 mice was similarly reduced following passive transfer of DENV-2 CRR vaccinated mouse sera (Hughes et al., 2012a). Together, these findings support the interpretation that the enhanced DHF-like disease and mortality of WT immunized mice and its significant reduction in pVD1-CRR vaccinated mice, was most likely due to the observed alterations of humoral immunity.

The pVD1-CRR vaccine exhibited an improved safety profile compared to WT in an *in vivo* AG129 model, however, some CRR vaccinated mice still succumbed to DENV disease, implying either that there could be additional pathogenic antibody classes not targeted by our CRR modifications or that mechanisms in addition to ADE may have been responsible for the mortality observed in pVD1-CRR vaccinated mice. Cross-reactive, non-neutralizing antibody recognizing prM has recently been reported to comprise a significant portion of the human anti-DENV response and such antibody can enhance DENV replication (Beltramello et al., 2010; Dejnirattisai et al., 2010; Rodenhuis-Zybert et al., 2010). However, the limited data suggest that prM antibodies may not be as frequently induced in mice as in humans (Henchal et al., 1985; Roehrig et al., 1998); this could be the result of different routes of infection, needle (i.m.) versus mosquito (i.d.) or of the different forms of virus (mature, immature, or partially mature) being delivered. Nevertheless, VLPs produced by our flavivirus and DENV plasmids in tissue culture do contain prM and vaccination with these plasmids can produce antibody recognizing prM (Chang et al., 2003; Chiou et al., 2012). CRR substitutions introduced into these plasmids do not alter the prM processing or change the relative ratios of VLP prM and E or their induced antibody populations in comparison to WT (Chiou et al., 2012). Thus, prM antibodies may be of concern in human vaccination and they could explain the residual enhancement observed in CRR vaccinated AG129 mice (Henchal et al., 1985; Roehrig et al., 1998), but they are unlikely to account for the differences in pathology and mortality between pVD1-CRR and -WT immunized mice in this study. Alternatively, residual CRR vaccinated mouse enhancement could have occurred via antibody recognizing E protein epitopes outside of the CRR modifications and/or from lower, non-protective levels of normally protective antibodies (Pierson et al., 2008; Beltramello et al., 2010; Hughes et al., 2012a).

Antibody-mediated neutralization and enhancement are two phenomena that exist along a continuum of antibody concentration. Antibody-mediated neutralization of flaviviruses requires virion loading with a stoichiometry that exceeds the neutralization threshold. This neutralization threshold varies for different antibodies based upon their affinity and epitope accessibility. When antibody occupancy does not exceed this neutralization threshold, enhancement can occur (Pierson et al., 2008). EDII_FP_ epitopes are relatively inaccessible on mature and partially mature virions and antibodies recognizing these epitopes typically require high to saturated occupancy if they are to neutralize virus at all. Such antibodies therefore have the potential to enhance infection across a wide range of concentrations. Serotype-specific potently neutralizing antibodies recognizing EDIII lateral ridge epitopes such as DENV-2 specific 3H5 and 9A3D-8 however, are able to neutralize virus at much lower occupancy thresholds (Pierson et al., 2008). The implication of these findings is that antibody recognizing EDII_FP_ epitopes will tend to enhance infection at most concentrations and only neutralize at very high concentrations whereas EDIII serotype-specific antibodies will neutralize across most concentrations and only enhance at the lowest concentrations. Thus, the reduced levels of cross-reactive EDII_FP_ recognizing antibody and increased levels of DENV-2 specific antibody in pVD1-CRR immunized mice supports their playing important roles in the observed differences in morbidity and mortality.

The concern of vaccine-induced immunodominant antibody responses in dengue vaccinology and our approach of genetically modifying these pathogenic epitopes to redirect the immunodominance hierarchy have parallels with other multi-strain pathogens such as HIV and Influenza. Both HIV and influenza vaccinology have to address original antigenic sin and strain-specific immunodominance. For all of these viruses, producing efficacious and improved next-generation vaccines is likely to require altering the native, WT immune responses (Ndifon et al., 2009; Miller, 2010; Durbin and Whitehead, 2011; Nara et al., 2011; Schmitz et al., 2011). In both HIV and influenza there has been interest in this area of modifying immunogens to redirect immune responses, typically referred to as immune dampening and immune refocusing. Some generalizations from this body of work are consistent with and support our CRR DENV DNA vaccination results. The dampening of immunodominant epitopes resulted in decreased induction of antibodies recognizing the targeted epitopes while increasing the amount of antibody stimulated from natively sub-dominant epitopes. Moreover, in spite of such major alterations in immunodominance hierarchies, antigenically modified immunogens induced similar total overall antibody titers as did WT immunogens (Tobin et al., 2008).

There is substantial interest in utilizing heterologous vaccine prime-boost strategies to improve and broaden immunogenicity, especially in the context of DNA vaccination (Dale et al., 2006; Chen et al., 2007; Simmons et al., 2010; Ding et al., 2011; Guenaga et al., 2011). In this context, “heterologous” typically refers to the use of different vaccine formats to present the same viral immunogens between prime and boost, most commonly DNA prime and protein (recombinant, inactivated virus, or live attenuated virus) boost. Much of this interest has also been directed toward HIV (Walker and Burton, 2010) and influenza (Wei et al., 2010; Ding et al., 2011) where the vaccine goal is to increase the breadth of neutralization and hence protection from these highly variable multi-strain pathogens. Because of the similarities to DENV and the difficulties of rapidly inducing balanced tetravalent immunity with current DENV live-attenuated vaccines (Guy et al., 2009; Murphy and Whitehead, 2011), DNA prime-protein boost strategies are particularly appealing to DENV vaccinology. We found that DENV-1 CRR DNA vaccination redirected subsequent immunity in response to DENV-2 challenge and increased the induction of a broad repertoire of neutralizing antibodies to produce a polyclonal DENV-2 neutralizing response with increased cross-neutralization to other DENV serotypes. Our findings suggest that CRR DNA vaccines hold potential for novel DENV vaccine strategies that take advantage of the benefits of both DNA and live attenuated virus vaccines. One such strategy is to prime hosts with a low-dose tetravalent CRR DNA vaccine to elicit a beneficial memory response with reduced enhancing capability and limited neutralizing antibody to each serotype. Such priming might allow for efficient tetravalent live-attenuated virus boost 1–2 months later to produce balanced and protective tetravalent immunity. We are currently initiating preclinical studies using this strategy with the goal to achieve protective tetravalent immunity within 3 months; a long sought after goal of DENV vaccinology that continues to elude existing DENV vaccine candidates.

## Conflict of Interest Statement

The findings and conclusions in this article are those of the authors and do not necessarily represent the views of the Centers for Disease Control and Prevention.
